# Predicting OCT biological marker localization from weak annotations

**DOI:** 10.1038/s41598-023-47019-6

**Published:** 2023-11-11

**Authors:** Javier Gamazo Tejero, Pablo Márquez Neila, Thomas Kurmann, Mathias Gallardo, Martin Zinkernagel, Sebastian Wolf, Raphael Sznitman

**Affiliations:** 1 Artificial Intelligence in Medical Imaging, University of Bern, 3008 Bern, Switzerland; 2 Department of Ophthalmology, Bern University Hospital, 3010 Bern, Switzerland

**Keywords:** Biomarkers, Computer science, Medical imaging

## Abstract

Recent developments in deep learning have shown success in accurately predicting the location of biological markers in Optical Coherence Tomography (OCT) volumes of patients with Age-Related Macular Degeneration (AMD) and Diabetic Retinopathy (DR). We propose a method that automatically locates biological markers to the Early Treatment Diabetic Retinopathy Study (ETDRS) rings, only requiring B-scan-level presence annotations. We trained a neural network using 22,723 OCT B-Scans of 460 eyes (433 patients) with AMD and DR, annotated with slice-level labels for Intraretinal Fluid (IRF) and Subretinal Fluid (SRF). The neural network outputs were mapped into the corresponding ETDRS rings. We incorporated the class annotations and domain knowledge into a loss function to constrain the output with biologically plausible solutions. The method was tested on a set of OCT volumes with 322 eyes (189 patients) with Diabetic Macular Edema, with slice-level SRF and IRF presence annotations for the ETDRS rings. Our method accurately predicted the presence of IRF and SRF in each ETDRS ring, outperforming previous baselines even in the most challenging scenarios. Our model was also successfully applied to en-face marker segmentation and showed consistency within C-scans, despite not incorporating volume information in the training process. We achieved a correlation coefficient of 0.946 for the prediction of the IRF area.

## Introduction

Age-Related Macular Degeneration (AMD) and Diabetic Retinopathy (DR) are two of the most common eye diseases, with over 300 million patients at risk of losing sight worldwide^1^. To diagnose and manage these chronic retinal conditions, 30 million Optical Coherence Tomography (OCT) are taken each year, yielding micron-resolution 3D volumes of the retina in a routine, fast, and noninvasive way. OCT has become a crucial instrument for establishing patient treatments and a dependable tool to validate the efficacy of novel therapeutic approaches to treat eye diseases.

**Figure 1:**
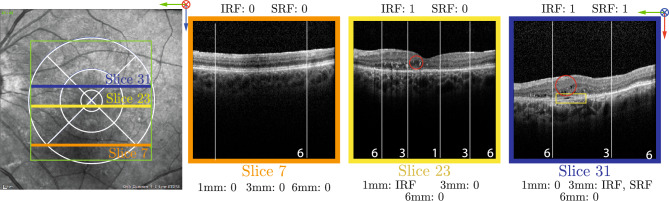
Left: View of the retina, the OCT volume (green square) and the ETDRS rings (white) which are virtually placed on the surface of the retina. Right: Three 2D OCT slices at different positions of the OCT volume. Red circles indicate IRF biological marker and the yellow rectangle indicates SRF (figure best seen in color).

In this context, intraretinal fluid (IRF) and subretinal fluid (SRF) are well-established markers that are directly linked to both AMD and DR^2,3^. Their identification and localization within a set of concentric rings, known as the Early Treatment Diabetic Retinopathy Study (ETDRS) rings^4^, is critical for disease assessments (see Fig. 1), as the different ETDRS ring regions are linked to different visual function levels (i.e., higher risk of vision loss when markers are in the central 1mm ring and lower risk when in the 6 mm ring). Driven by this clinical need, numerous methods have been proposed to automate the process of identifying markers such as IRF and SRF^5^, and the work here follows this research direction too.

Previous methods have included IRF and SRF detection and segmentation models^6–9^. While segmentation models have the advantage of quantifying IRF and SRF regions, they often require a large amount of manually annotated segmentation labels for optimal performance. To counteract this issue, some works use weak annotations, such as slice level labels, retinal layer positioning, and foveal distance, to achieve voxel-wise segmentations^10^. Weak annotations offer a wide range of possibilities, and therefore others have studied the use of bounding boxes to develop positive-aware lesion detection networks^11^. More relevant to our work, some methods only use slice-level annotations^12^. Here, Ma et al. presented a weakly-supervised segmentation method for Geographic Atrophy (GA) lesions in Spectral Domain OCT images. The method first segments the retinal pigment epithelium and then extracts a class activation map from multi-scale features. The final en-face binary segmentation of GA is obtained by refining the map with Conditional Random Fields, utilizing only slice-level labels with binary information about the presence of GA.

Similarly, ensembles of Convolutional Neural Networks (CNNs) have been proposed to detect IRF and SRF in individual slices using only binary annotations on a slice level^13,14^. However, by removing the need for segmentation annotations, these methods cannot provide any location information. In this work, we propose a novel weakly supervised deep learning framework that overcomes these limitations and enables the detection and localization of 2D OCT markets in ETDRS rings without requiring costly location information during training. Specifically, our method uses binary annotations of marker presence in OCT slices during training and infers marker presence and marker location in ETDRS rings during test time. To do this, we introduce a pooling strategy where we treat our network’s convolutional feature maps in such a way as to preserve spatial relations that can be partially pooled for coarse localization. This is combined with a novel loss function that enforces geometrically and biologically plausible solutions. This allows ring assignment to be performed as a post-processing step independent of the training phase. Our experiments demonstrate that our method predicts the location of markers in ETDRS rings with high accuracy, thereby significantly outperforming previous methods that use the same amount of training information.

## Methods

Our objective is to train a method capable of inferring in which ETDRS ring different markers are located, but only using 2D OCT slices and associated slice-level binary annotations. In a 2D OCT slice, ETDRS rings correspond to a set of non-continuous vertical stripes (see Fig. 1). From the placement of the ETDRS rings on the OCT volume, we make the following three important observations: (1) depending on where an OCT slice is positioned in the volume, different ETDRS rings are visible in the slice, (2) the width of different rings depends on where an OCT slice is positioned and (3) ring symmetry is preserved regardless of the slice position. We will explicitly leverage these observations to design and train our approach.

**Figure 2:**
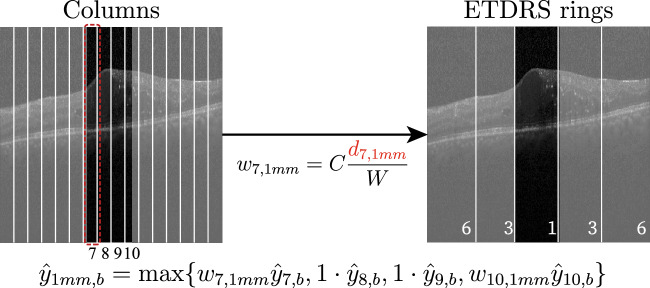
Mapping column predictions to ring predictions for the central slice of an OCT volume. The column layout (left) is shared among all slice positions. The ring layout (right) is specific to the slice location in the volume. $w_{i,j}$ is the contribution of ring *j* in the *i*-th column and $\hat{\textbf{y}}_{i,b}$ is the prediction for ring *i* and marker *b*.

Specifically, instead of training our method to produce different outputs depending on the slice location, we predefined a partition of 2D OCT slices into image columns (see Fig. 2 left). That is, we will train our method to produce predictions for each of these columns, regardless of the specific slice location within the volume. At the end of this section we describe the straightforward post-processing mapping from column-level predictions to the ETDRS rings (as shown in Fig. 2 left).

### Model

Formally, we partition a 2D OCT slice, $\textbf{x}$, into *C* equally spaced columns. We wish to train a model $f:[0,1]^{H\times W} \rightarrow [0,1]^{(1+C)\times B}$ that maps $\textbf{x}$ to a collection of probabilities $\hat{\textbf{y}}$, where *B* is the number of different possible types of markers to be found. For each marker $b\in B$, the collection $\hat{\textbf{y}}$ contains both the probability of presence of *b* in the entire OCT slice, $\hat{\textbf{y}}_{0,b}$, and the probability of presence of *b* in each column $c\in C$, denoted $\hat{\textbf{y}}_{c, b}$. Our training data is made of tuples $(\textbf{x}, \textbf{y}_0)$, with OCT slice $\textbf{x}$ and corresponding slice-level annotations $\textbf{y}_0\in \{0, 1\}^B$ with no reference whatsoever to the ring or column in which they are located. A comprehensive list of all the variables can be found in Table S1 in the supplementary material.﻿

**Figure 3:**
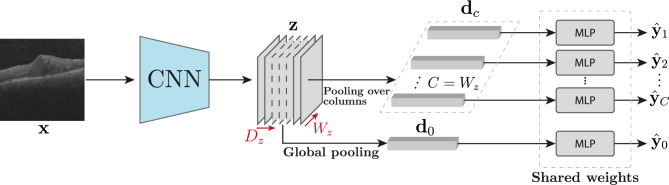
Our proposed network architecture: usage of partial pooling to extract information from the feature map to infer location outputs with a set of shared-weight MLPs.

Figure 3 depicts our model architecture. The input OCT slice is processed by a CNN which produces a feature map $\textbf{z}\in \mathbb {R}^{D_z\times H_z\times W_z}$ with width equal to the number of columns $C = W_z$. We then apply a number of pooling operations over the feature map $\textbf{z}$ to describe the entire OCT slice as well as every column *c*. In particular, to identify markers that may appear as large or small in a given image, we set the descriptor of the entire OCT slice to be a $2D_z$-dimensional vector $\textbf{d}_0=[\mathop {\mathrm {avg\_pool}}\limits (\textbf{z}), \mathop {\mathrm {max\_pool}}\limits (\textbf{z})]$ obtained as the concatenation of average pooling and maximum pooling over the spatial dimensions of $\textbf{z}$. Likewise, the descriptor of every column *c* is another $2D_z$-dimensional vector $\textbf{d}_c=[\mathop {\mathrm {avg\_pool}}\limits (\textbf{z}_{\cdot ,\cdot ,c}), \mathop {\mathrm {max\_pool}}\limits (\textbf{z}_{\cdot ,\cdot ,c})]$ obtained as the concatenation of the two pooling operators acting on the corresponding column of $\textbf{z}$. The descriptor vectors are then processed by a multi-layer perceptron (MLP) followed by an element-wise sigmoid activation to produce the final probabilities,

1 $$\begin{aligned} \hat{\textbf{y}}_0 = \sigma \left( \text {MLP}(\textbf{d}_0)\right) ,\quad \quad \hat{\textbf{y}}_c = \sigma \left( \text {MLP}(\textbf{d}_c)\right) \quad \forall c. \end{aligned}$$

### Training

 We use a combination of three loss terms to train our model. The first term uses the standard binary cross entropy (BCE) of the slice-level predictions $\hat{\textbf{y}}_0$ with the slice-level ground-truth annotations $\textbf{y}_0$,

2 $$\begin{aligned} \ell _1(\hat{\textbf{y}}, \textbf{y}_0) = \sum _{b} \text {BCE}(\hat{\textbf{y}}_{0,b}, \textbf{y}_{0,b}). \end{aligned}$$

The second term incorporates constraints on column-level predictions based on the image-level ground-truth. Specifically, when a biological marker is not present in the input image, $\textbf{y}_{0,b} = 0$, we penalize high predicted probabilities for *b* in all the columns. On the other hand, if the marker is present, $\textbf{y}_{0,b}=1$, we encourage a high probability for *b* for at least one column. Formally, we compute,

3 $$\begin{aligned} \ell _2(\hat{\textbf{y}}, \textbf{y}_{0,b}) = -\sum _b (1-\textbf{y}_{0,b})\dfrac{1}{C}\sum _c \log (1-\hat{\textbf{y}}_{c,b}) - \sum _b \textbf{y}_{0,b}\max _c \log \hat{\textbf{y}}_{c,b}. \end{aligned}$$

The last term imposes invariance to horizontal symmetry on the column-level probabilities. When our model receives a horizontally flipped image $\textbf{x}'$, the predicted column-level probabilities $\hat{\textbf{y}}'$ should also be flipped, and therefore $\hat{\textbf{y}}_{c,b}$ should be equal to $\hat{\textbf{y}}'_{C-c,b}$ for all *b*. To this end, we penalize a symmetric KL divergence between the corresponding probabilities,

4 $$\begin{aligned} \ell _3(\hat{\textbf{y}}, \hat{\textbf{y}}') = \dfrac{1}{2}\sum _{c, b} \left( D_{KL}\big ({\hat{\textbf{y}}_{c, b}}\Vert {{\hat{\textbf{y}}'_{C-c, b}}}\big ) + D_{KL}\big ({\hat{\textbf{y}}'_{c, b}}\Vert {{\hat{\textbf{y}}_{C-c, b}}}\big ) \right) . \end{aligned}$$

**Figure 4:**
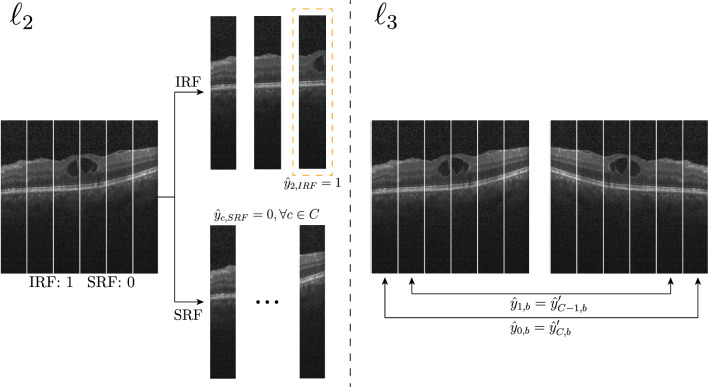
Graphical explanation for $\ell _2$ and $\ell _3$ in a slice where IRF is present and SRF is not. In this example, with $\ell _2$, we enforce that IRF must be present in at least one column while SRF is not found anywhere. With $\ell _3$ we incorporate symmetry consistency among the flipped and the non-flipped slices.

Specifically, $\ell _3$ incorporates the symmetry of the ETDRS rings we wish to induce in our model. Note that the desired horizontal symmetry cannot be obtained by random horizontal image flipping augmentation, however, as $\ell _3$ enforces predictions on the columns to be consistent regardless of whether the image is flipped or not. Using a similar symmetry argument for $\ell _1$ and $\ell _2$, our final loss is,

5 $$\begin{aligned} \mathscr {L}(\hat{\textbf{y}}, \hat{\textbf{y}}', \textbf{y}_0)= & {} \ell _1(\hat{\textbf{y}}, \textbf{y}_0) + \ell _1(\hat{\textbf{y}}', \textbf{y}_0) + \ell _2(\hat{\textbf{y}}, \textbf{y}_0) + \ell _2(\hat{\textbf{y}}', \textbf{y}_0) + \ell _3(\hat{\textbf{y}}, \hat{\textbf{y}}'), \end{aligned}$$

where $\hat{\textbf{y}}$  and $\hat{\textbf{y}}'$ are the predicted probabilities for the input image $\textbf{x}$ and corresponding horizontally-flipped version $\textbf{x}'$, respectively. Figure 4 shows a graphical explanation for $\ell _2$ and $\ell _3$.

### Inference

At test time, we can infer the layout of ETDRS rings in an OCT slice once a slice is evaluated by our network. This correspondence is not one-to-one, as a single ring usually contains several columns, and one column may be shared between two rings. To thus produce ring-level predictions, we compute the maximum of the probabilities of the columns contained in each ring. For columns spanning two rings, we weigh the contribution of the column by the proportion of the column inside each ring, as shown in Fig. 2.

## Results

We present the descriptive statistics of the training and testing sets in Table 1. The OCT data comes from the Dept. of Ophthalmology, Bern University Hospital (Switzerland) and was acquired using the Heidelberg Spectralis system. The resolution of all slices is 496 × 512 pixels. The training and testing sets are similar in terms of pathologies, with the main difference being the granularity of the annotations: the training dataset only contains slice-level annotations for two biological markers, while the testing dataset includes additional ETDRS rings information at 1 mm, 3 mm and 6 mm per slice. In addition, a subset of the testing dataset has been manually segmented. Therefore, pixel-wise annotations for IRF and SRF are also available in 54 volumes (2646 OCT slices). None of the patients in this test data are present in the training data. The distribution of IRF and SRF occurrences in the testing dataset is given in Table 2.

**Table 1:** Dataset description.

	Training dataset	Testing dataset
Number of patients	433	189
Number of eyes	460	322
Number of slices	22,723	28,322
Present pathologies	Diabetic retinopathy with and without Diabetic Macular Edema (DME), and early, intermediate, and late AMD	DME
Annotations	Slice-level annotations for IRF and SRF	Slice-level SRF and IRF presence annotations for the ETDRS rings at 1 mm, 3 mm, and 6 mm

### Implementation and baselines

The backbone model of our method is an EfficientNet-b4^15^ with ImageNet-initialized weights. As a preliminary step, we train the network alone in the task of IRF and SRF multilabel classification to produce slice-level predictions, and then fine-tune the entire model as described in the Methods section with the loss function of Eq. (5). We use a batch size of 32 slices, SGD with momentum of 0.9 and a base learning rate of $5\cdot 10^{-3}$ which is scaled by 0.99 after every epoch. The feature map z of EfficientNet-b4 is sized 1792 × 16 × 16. We perform maximum and average pooling per column to produce C = 16 descriptor vectors $d_c$ of dimension $2D_z=3584$, which are subsequently processed by the MLP to get 16 column-level predictions. The MLP itself consists of a single linear layer with 2 outputs for SRF and IRF, followed by a sigmoid activation. The column-level predictions are then mapped to ring-level predictions as explained at the end of the Methods section.

While there are no direct existing baselines for localization of OCT biological markers with weak annotations, we compare our method to the following alternative baselines:

- *Masking* At test time, we mask the slice regions to only reveal relevant ETDRS rings and feed this to an EfficientNet trained on the slice-level detection task (as above). This masking has been done by replacing all pixels in the region to 0.
- *Masking with partial convolutions (PartConvs)* As in the *Masking* baseline but replacing all convolutional layers by partial convolutions^16^, except for those in the squeeze and excitation blocks^17^, so to ignore masked regions.
- *Grad-CAM* We use Grad-CAM^18^ to build a 16 × 16 heatmap for each output variable and pick the maximum value of each column. This serves as a column-level measurement of the presence of SRF and IRF. We use the pre-trained EfficientNet to obtain the final ring-level predictions by applying the same post-processing mapping explained at the end of the Methods section.
- *MS-CAM*^12^ this approach consists of two stages: first, the activations of the different features of the resolutions are combined with Grad-CAM++ to obtain a pixel-wise segmentation. Second, these segmentations are refined using CRF on the en-face projection image. We reproduced the first stage and converted the resulting pixel-wise segmentation into rings.

All methods were implemented using PyTorch. Our method and the baselines were trained for 10 epochs.

### Localization results

**Figure 5:**
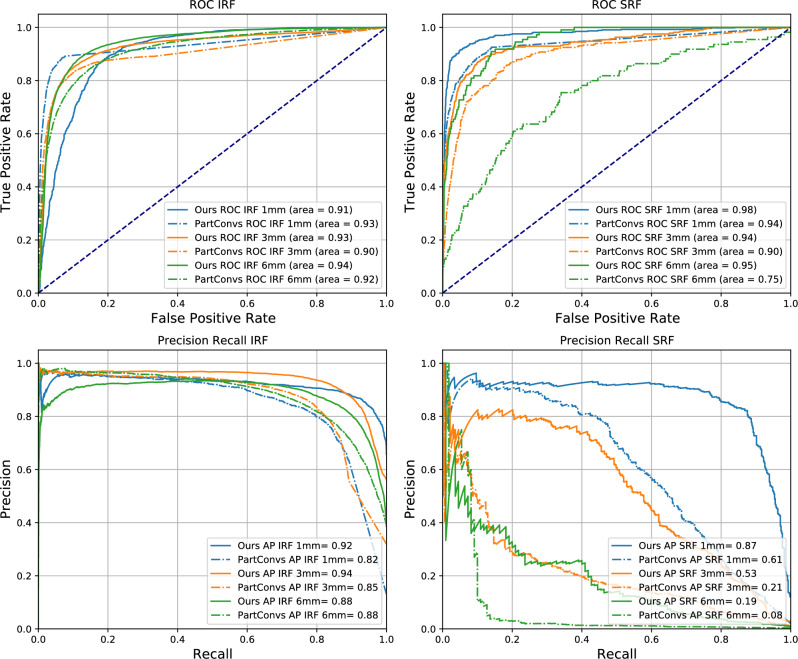
ROC and Precision-Recall curves for both markers and rings with our proposed method on the testing dataset (solid lines). Results are compared to Partial Convolutions (dashed lines) on the same dataset.

Table 2 reports the performance of all methods in terms of AUC ROC and Average Precision (AP). Our method achieves the highest ROC-AUC and AP for every marker and ETDRS ring. The improvement is particularly notable for 6 mm SRF, where our method doubles the performance of other baselines in AP. Figure 5 compares our method’s ROC and Precision-Recall curves and the PartConvs baseline throughout the three ETDRS rings. Table 2 also shows the occurrence of both biological markers in each one of the rings. IRF is present in 51.5% of the images in the testing set, while SRF is scarcer and present in only 2.8%. This imbalance is further exacerbated in the ring annotations: as depicted in Fig. 1, where the 6 mm ring is present in all the B-Scans. However, 38.4% have IRF in the 6 mm ring, but only 0.4% have SRF. This is explained by the fact that SRF is unlikely to be found in the outer rings, leading to a lower number of occurrences in the test set than IRF.

**Table 2:** Comparison of the proposed method to evaluated baselines in terms of AUC ROC and AP on the Location dataset for all markers on the entire slice and in the different ETDRS rings.

		1 mm		3 mm		6 mm		Present	
		IRF	SRF	IRF	SRF	IRF	SRF	IRF	SRF
	Occ. (%)	13.0	2.1	31.9	1.2	38.4	0.4	51.5	2.8
AUC	Masking	92.6	91.7	89.6	81.6	92.7	66.7	96.5	96.2
	PartConvs	**93.2**	94.1	90.2	89.8	91.7	74.9	94.2	89.7
	Grad-CAM	85.2	87.3	89.2	76.0	89.4	64.6	96.5	96.2
	MS-CAM^12^	55.7	70.4	57.5	64.1	55.0	56.0	56.0	53.7
	Ours	90.6	**97.5**	**92.7**	**93.8**	**94.1**	**95.1**	**97.2**	**97.7**
AP	Masking	76.9	48.3	84.2	17.2	**88.5**	5.6	96.1	72.3
	PartConvs	81.8	60.5	85.2	21.4	88.1	8.2	94.2	38.5
	Grad-CAM	88.2	68.4	86.9	25.4	78.7	7.4	96.1	72.3
	MS-CAM^12^	70.3	38.5	64.1	14.4	54.3	1.6	55.6	3.1
	Ours	**92.1**	**86.6**	**93.7**	**52.7**	88.3	**19.1**	**96.8**	**77.9**

The first row indicates the occurrences of each marker in this dataset.

Best performing method for each biological marker is marked in bold.

**Figure 6:**
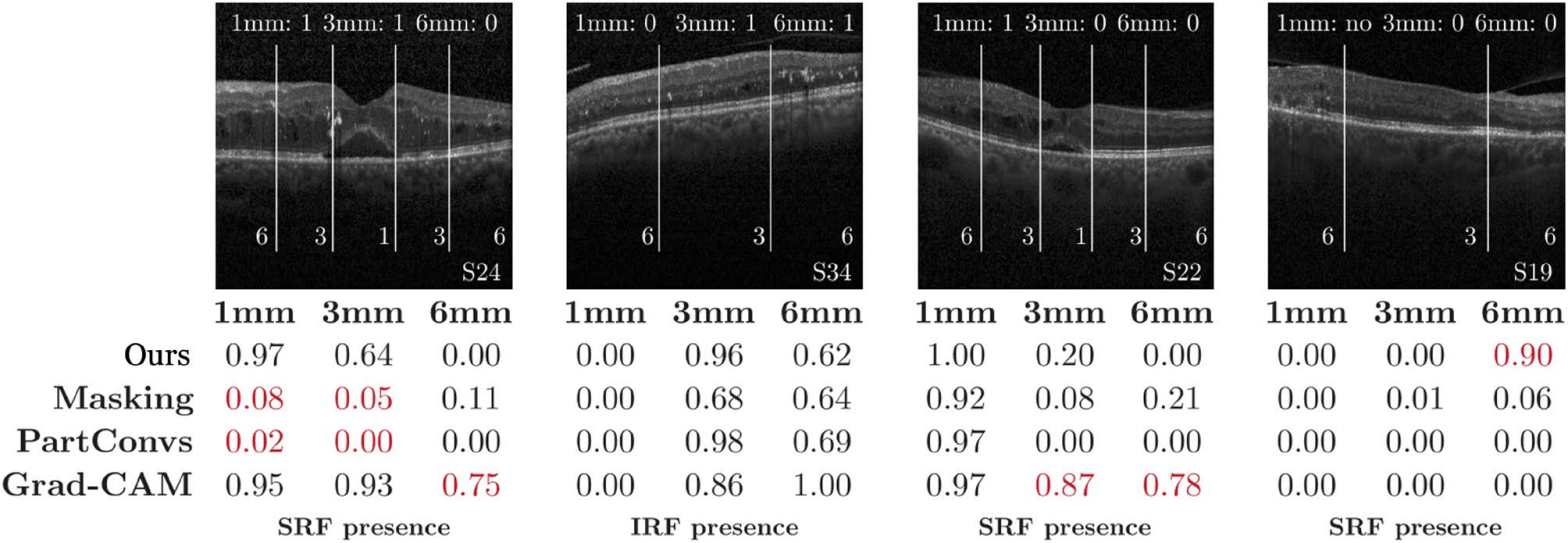
Outputs of our method and baselines on four examples. In each OCT image, we show the slice number (bottom right) and in which ring the marker can be found (top row). We highlight incorrect detections in red.

Figure 6 illustrates the performance of the different methods in several cases. We provide additional cases in Fig. S1 in the supplementary material.

### Segmentation results

To further demonstrate the accuracy of our method in locating biological markers, we also compare our results to the subset of test images for which IRF and SRF ground-truth segmentations are available (2’646 OCT slices). For this purpose, a column is considered positive for a marker if it contains at least one pixel of that marker. Our method achieved AUCs over 90% for both IRF and SRF, as shown in Table 3. The low mAP for SRF can be attributed to its very low occurrence rate.

**Table 3:** Results on the segmentation dataset.

	IRF	SRF
ROC AUC	91.1	93.7
mAP	81.2	64.8

### En-face projection

Before the post-processing step that converts columns to rings, our method produces a coarse 1D segmentation per B-Scan. The projection of this output and further concatenation of all the B-Scans that compose a C-Scan results in the en-face projection.

In Table 4, we compared the coarse en-face projections that our method produces to the 54 manually segmented volumes and computed the mean area of IRF and SRF. We used a resolution of $11.72\; \upmu \text{m}/\text{px}$ and $120\; \upmu \text{m}/\text{slice}$ in the lateral and sagittal axes respectively. The row “Expert” refers to pixelwise segmentations, and “16 column Expert” has been calculated by converting the pixelwise segmentation into columns, with the same methodology as in the previous section. We believe “16 column Expert” version is a fairer comparison because it provides the same amount of information as our predictions. For IRF, we obtain a mean area of $5.73\; \text{mm}^2$, being $6.29\; \text{mm}^2$ the ground truth with 16 columns. For SRF, this number is less representative since only 25 of the 54 volumes contain this type of fluid.

**Table 4:** En-face projection results.

	IRF (mm^2^)	SRF (mm^2^)
Expert	$3.15\pm 2.97$	$0.21\pm 0.63$
16 column expert	$6.29\pm 4.93$	$0.35\pm 0.89$
Predicted	$5.73\pm 5.71$	$0.12\pm 0.30$
MSE: predicted vs 16 column expert	$3.89\pm 8.90$	$0.46\pm 2.11$

**Figure 7:**
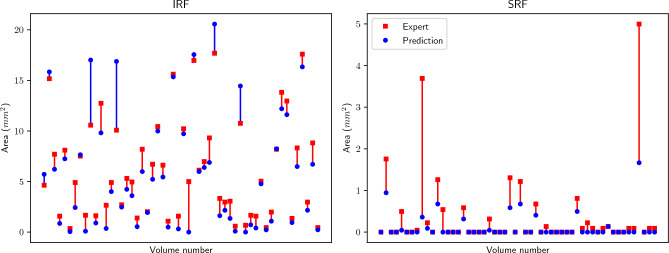
Expert (red) and predicted (blue) area for IRF and SRF in each one of the 54 volumes. Blue (red) vertical lines refer to overestimations (underestimations) of our model with respect to the Expert segmentation. Volumes are sorted in decreasing order of SRF area discrepancy, and that sorting is kept for IRF.

**Figure 8:**
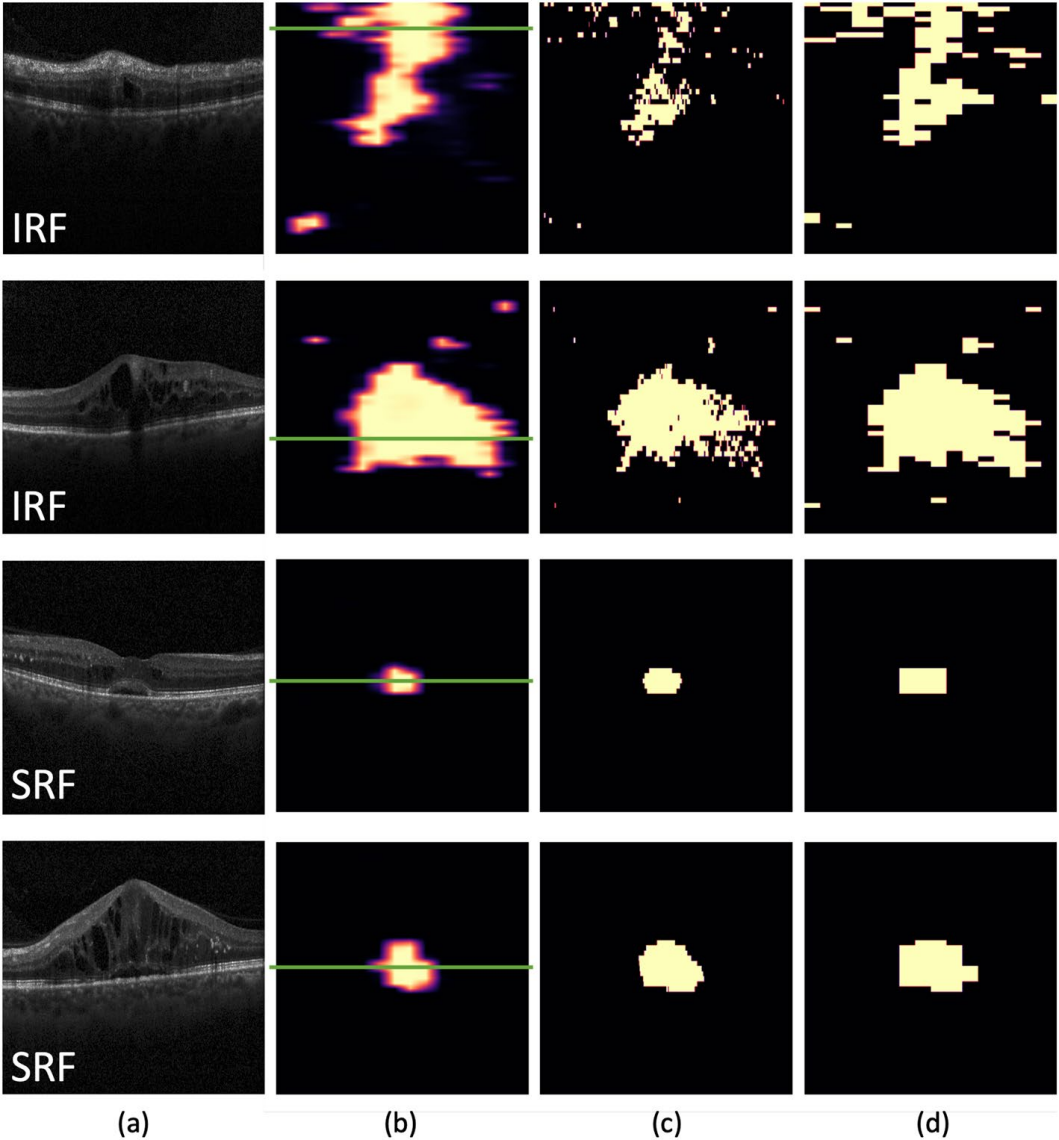
En-face projection results for the specified markers. (**a**) B-Scan at the location in green. (**b**) Prediction with our method. Different colors represent the uncertainty of the model. Lighter means more certain. (**c**) Expert pixelwise segmentation. (**d**) Expert segmentation converted into columns. Figure best seen in color.

Figure 7 shows the comparison of the predicted area per volume (blue) and the area delimited by the expert (red). Both figures have been calculated using the column system. Figure 8 shows qualitative results of four of the volumes. Here, we compare the expert en-face projection with full segmentation (c) and after column conversion (d) to our prediction (b).

**Figure 9:**
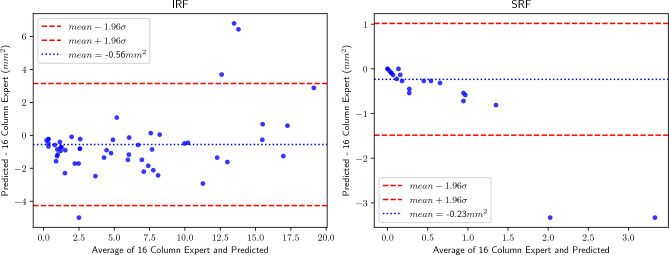
Bland–Altman plots for IRF and SRF.

To assess the clinical relevance of our method, understood as the agreement between our approach and an expert-based segmentation, we built Bland-Altman plots for SRF and IRF segmentations. In Fig. 9, we compared our prediction for the coarse en-face segmentation to the 16-column Expert in each volume. We see that four volumes fall outside one standard deviation for IRF, while only two in the case of SRF.

**Figure 10:**
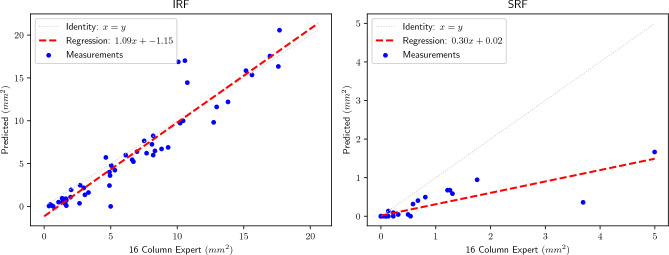
Correlation plots for IRF and SRF.

Figure 10 shows correlation plots for both biomarkers. For IRF, the linear regression returns $R^2=0.895$ and a slope close to identity (1.09). On the contrary, for SRF our method achieves $R^2=0.760$ and a slope of 0.30.

### Ablation results

We conduct an ablation study to quantify how the different loss terms in Equation 5 affect the method’s performance. Table 5 shows the AUC ROC and AP of all predicted outputs as a function of what loss terms are included when training. The first and fourth rows, labeled with $l_1$, correspond to using the traditional Cross Entropy loss, which does not make use of any column constraint. $l_2$ and $l_3$ do impose these constraints as described in Method. On average, there is an improvement of 5.4% ROC AUC (18.0% AP) after the addition of column constraints, which is further increased to 7.6 and 19.1%, respectively, with horizontal symmetry.

**Table 5:** Ablation study. We quantify the performance of our method when using using only the terms {$\ell _1$}, {$\ell _1$, $\ell _2$} or {$\ell _1$, $\ell _2$, $\ell _3$} in our loss functions.

		1 mm		3 mm		6 mm		Present	
		IRF	SRF	IRF	SRF	IRF	SRF	IRF	SRF
AUC	$\ell _1$	88.0	90.7	90.2	81.9	92.6	73.7	96.5	96.2
	$\ell _1, \ell _2$	87.8	96.9	90.1	92.3	92.2	91.7	96.5	95.9
	$\ell _1, \ell _2, \ell _3$	**90.6**	**97.5**	**92.7**	**93.8**	**94.1**	**95.1**	**97.2**	**97.7**
AP	$\ell _1$	91.5	71.4	91.7	29.4	85.8	13.8	96.1	72.3
	$\ell _1, \ell _2$	90.9	84.8	91.7	41.5	86.5	**24.4**	96.3	77.1
	$\ell _1, \ell _2, \ell _3$	**92.1**	**86.6**	**93.7**	**52.7**	**88.3**	19.1	**96.8**	**77.9**

Significant values are in bold.

## Discussion

The proposed method achieved satisfactory results in all ETDRS rings and studied biological markers. Our method outperforms the compared baselines for every marker and ETDRS ring, confirming our hypothesis that feature maps can be used to coarsely identify marker locations.

We note, however, that, in terms of AP, the prediction performance for SRF in the 3 mm and 6 mm rings is significantly lower than other reported values for all methods. As discussed in the Results section, it is unlikely to find SRF in the outer rings, leading to a lower number of occurrences of this biological marker. This in turn strongly reduces the precision of the methods as soon as there are just a few false positive detections. The associated AUC ROC scores do not exhibit this behavior since they include false positive rates.

The segmentation results in Table 3 show that the proposed method is robust even before our post-processing mapping for coarse biomarker localization, meaning that the post-processing step is transparent to the performance of the model.

The ablation studies in Table 5 suggest that not only is the architecture itself important ($l_1$) but so is enforcing coherence in the column outputs with the slice labels (i.e. $l_2$). In the case of SRF at 3 mm and 6 mm, this provides a significant performance increase without reducing the performances of other outputs. For SRF, AP increases by 41.2% at 3 mm and 43.4% at 6 mm, while close to no difference is seen for IRF, where the results with $l_1$ already outperform some of the baselines. This boost at the outer rings is highly beneficial as the presence of biological markers in these rings (especially SRF) is highly scarce, therefore making it harder to train appropriately for. In our testing dataset, acquired with a variety of eyes and patients, only 113 out of 28’322 B-Scans showed SRF in the 6 mm ring. Finally, including $l_3$ brings modest but consistent gains when $l_1$ and $l_2$ are already used.

Compared to other methods, our model is more robust than the baselines, giving more reliable results even in hard situations where previous methods struggle. Grad-CAM is the go-to method in virtually all weakly supervised segmentation methods for OCT, which rely on a CAM-based approach with various architectures. In this line, MS-CAM^12^ proposes an architecture that leverages the activations of the different feature resolutions of the backbone and then combines them with Grad-CAM++. Segmentations are then refined using CRF on the en-face projection image. For a fair comparison with our method, which does not use C-Scan information, we implemented only the first stage. The results (Table 2 show a performance worse than other baselines, with a strong difference in more difficult scenarios. For SRF at the 6 mm ring, MS-CAM achieves 1.6 AP, compared to 19.1 with our method. We believe that CRF refinement plays a major role in MS-CAM by reducing the over-segmentation produced by the first stage. Over-segmentation leads to false positive predictions in our set-up, which reduces Average Precision.

Szeskin et al.^19^ use vertical pixel-wide columns in OCT slices to classify atrophic regions. Each B-Scan is partitioned into columns and fed with contiguous slices into a convolutional neural network, which outputs a binary label. The results are projected onto the infrared imaging image and are used to identify and segment atrophy lesions. Although this work looks similar to ours, it differs in the training scheme: while we only use slice-level annotations, allowing us for independent coarse segmentation per slice; the method proposed by Szeskin et al.^19^ uses per volume labeling in the form of IR image segmentation. Schlegl et al.^10^ focuses on voxel-wise segmentation and, while their output could be used for location as well, the method differs in intent and uses voxel-wise ground truth labels to train. Because our method uses only 2D slices and much weaker annotations, we believe it is not a comparable baseline.

Finally, the analysis of the en-face projection and segmentation results in Table 3, as well as Figs. 9 and 10, shows two aspects: (1) The outperformance of our model over the baselines does not depend on the post-processing step and (2) although both the training and inference act per B-Scan, the method is reliable and consistent when applied to all the slices in a volume. The Bland–Altman plot in Fig. 8, along with the correlation plot in Fig. 10, show that our area predictions per volume strongly agree with the Expert segmentation in the case of IRF. For SRF, this task remains challenging, and our method tends to underestimate the en-face area, as proved by the slope of 0.30 in the linear regression and the corresponding Bland-Altman plot. However, the end goal of our method is not to have an accurate segmentation of the en-face, but it comes as a byproduct.

We demonstrated that slice-level labels are sufficient to locate biological markers in ETDRS rings for OCT scans if weak constraints are enforced on the loss function. Furthermore, we confirmed that it is possible to modify the pooling strategy of a standard convolutional network to perform coarse localization without annotations. The method has proven to be more reliable than other baselines, even in hard situations where the number of training samples is scarce, as shown in Table 2. The ablation experiments in Table 5 demonstrate that the new terms in the loss function, especially $l_2$, are key to the performance of the model, producing consistent gains in all scenarios. Moreover, even if our method has only been presented with individual B-Scans during training, with no sense of complete volumes, it is capable of outputting volume-wise consistent predictions, as depicted in the segmentation and en-face projection results (Table 3 and Table 4 respectively). Lastly, there is no constraint in the loss function with regard to the markers that can be located. Therefore, the described approach could potentially be used to locate any biological marker as long as class labels are available.

Our approach has some limitations. Most notably, the granularity of the output before post-processing is constrained by the resolution of the feature maps. A more granular output would most likely improve the precision of the method. However, achieving such high-resolution feature maps collides with the main intention of classification neural networks, which are conceived to reduce the dimensionality of the inputs before the final linear layer. Another limitation comes from the variety of biological markers that have been tested. Due to labeling capacity and present pathologies in the data, only IRF and SRF were tested. Although the proposed method is agnostic to this aspect and potentially should behave equally with a larger cohort of markers, a more detailed study is required to confirm it.

## Conclusion

We have presented a method to locate markers in ETDRS rings for OCT scans by relying solely on slice-level annotations. By enforcing weak constraints on the loss function and modifying the pooling strategy of a standard convolutional network, we show that our method can learn to localize coarsely without annotations. To our knowledge, no other work has done so in the context of retinal imaging, and we have demonstrated that our approach achieves significant performance improvements over straightforward and state-of-the-art baselines. Further research will be focused on extending this to obtain per-pixel segmentation.

### Supplementary Information

Supplementary Information.

## Data Availability

The datasets generated during and/or analyzed during the current study are not publicly available as they are part of ongoing studies but are available subject to terms and conditions from the corresponding author on reasonable request.
