# Supplementary material for: Predicting OCT biological marker localization from weak annotations
Source: Sci Rep. 2023 Nov 11;13:19667. doi: 10.1038/s41598-023-47019-6 (PMC10640596; doi:10.1038/s41598-023-47019-6)
Supplement: Supplementary file 1 — Supplementary Information. [file 41598_2023_47019_MOESM1_ESM.pdf]

List of variables

Variable	Description
H	Height of the 2D slice
W	Width of the 2D slice
C	Number of columns the 2D slice is partitioned into
$\mathbf{x}$	2D OCT slice
$\mathbf{x}'$	Flipped 2D OCT slice
$\hat{\mathbf{y}}$	Collection of predicted probabilities for image $\mathbf{x}$
$\hat{\mathbf{y}}'$	Collection of predicted probabilities for the flipped image $\mathbf{x}'$
$\hat{\mathbf{y}}_{0,b}$	Probability of presence of b in the entire OCT slice
$\hat{\mathbf{y}}_{c,b}$	Probability of presence of b in column c
$\mathbf{y}_0$	Slice-level annotations
$\mathbf{y}_{0,b}$	Slice-level annotations for biomarker b
$\mathbf{z}$	Feature map after the backbone
$\mathbf{d}_0$	Descriptor of the entire OCT slice $\mathbf{d}_0 = [\text{avg\_pool}(\mathbf{z}), \text{max\_pool}(\mathbf{z})]$
$\mathbf{d}_c$	Descriptor of a column c, $\mathbf{d}_c = [\text{avg\_pool}(\mathbf{z}_{\cdot,\cdot,c}), \text{max\_pool}(\mathbf{z}_{\cdot,\cdot,c})]$

Table S1. List of variables and its description

Additional examples

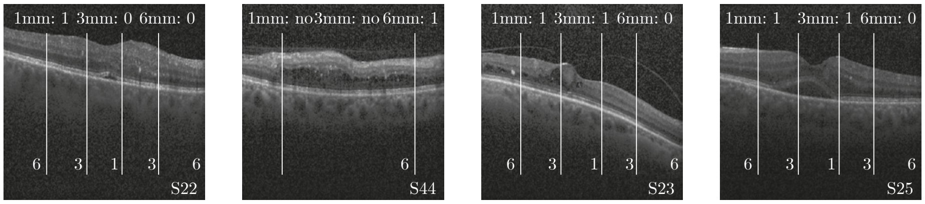

	1mm	3mm	6mm	1mm	3mm	6mm	1mm	3mm	6mm	1mm	3mm	6mm
Proposed	1.00	0.11	0.00	0.00	0.00	1.00	0.88	0.95	0.64	0.77	1.00	0.00
Masking	0.69	0.06	0.05	0.00	0.00	1.00	0.38	0.46	0.27	0.14	0.17	0.05
PartConvs	0.42	0.00	0.00	0.00	0.00	1.00	0.89	0.47	0.16	0.00	0.52	0.00
Grad-CAM	0.97	0.87	0.78	0.00	0.00	1.00	1.00	0.76	0.67	1.00	0.95	0.79

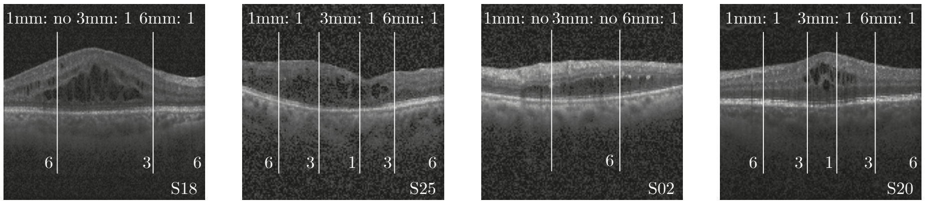

	1mm	3mm	6mm	1mm	3mm	6mm	1mm	3mm	6mm	1mm	3mm	6mm
Proposed	0.00	1.00	1.00	1.00	1.00	0.97	0.00	0.00	1.00	0.38	1.00	0.01
Masking	0.00	0.95	0.30	0.27	0.72	0.74	0.00	0.00	0.98	0.32	0.66	0.07
PartConvs	0.00	0.96	0.88	1.00	1.00	0.94	0.00	0.00	1.00	0.94	0.92	0.00
Grad-CAM	0.00	0.87	0.98	0.88	1.00	0.85	0.00	0.00	0.00	0.37	1.00	0.31

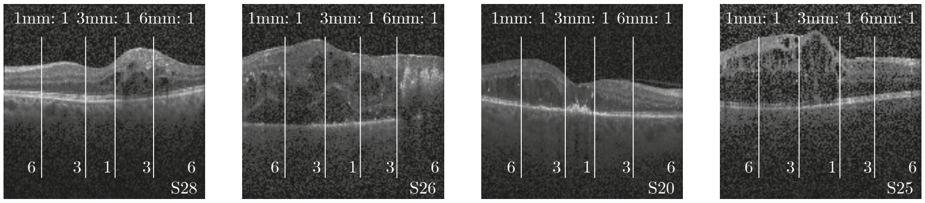

	1mm	3mm	6mm	1mm	3mm	6mm	1mm	3mm	6mm	1mm	3mm	6mm
Proposed	0.38	1.00	1.00	0.07	0.15	0.01	0.10	0.96	1.00	1.00	0.99	0.88
Masking	0.35	0.63	0.97	0.03	0.05	0.03	0.23	0.34	0.73	0.07	0.14	0.26
PartConvs	0.81	1.00	0.26	0.00	0.05	0.00	0.34	0.85	0.94	0.45	0.72	0.88
Grad-CAM	0.29	0.84	1.00	0.00	0.00	0.00	0.94	1.00	0.97	0.95	0.89	0.65

Figure S1. Outputs of our method and baselines on OCT slices. We show the slice number (bottom right) and in which ring the marker can be found (top row). We highlight incorrect predictions in red.
